# The Microbial Opsin Homolog Sop1 is involved in *Sclerotinia sclerotiorum* Development and Environmental Stress Response

**DOI:** 10.3389/fmicb.2015.01504

**Published:** 2016-01-07

**Authors:** Xueliang Lyu, Cuicui Shen, Yanping Fu, Jiatao Xie, Daohong Jiang, Guoqing Li, Jiasen Cheng

**Affiliations:** ^1^State Key Laboratory of Agricultural Microbiology, Huazhong Agricultural UniversityWuhan, China; ^2^The Provincial Key Lab of Plant Pathology of Hubei Province, College of Plant Science and Technology, Huazhong Agricultural UniversityWuhan, China

**Keywords:** *Sclerotinia sclerotiorum*, microbial opsin, sclerotial development, virulence, stress response

## Abstract

Microbial opsins play a crucial role in responses to various environmental signals. Here, we report that the microbial opsin homolog gene *sop1* from the necrotrophic phytopathogenic fungus *Sclerotinia sclerotiorum* was dramatically up-regulated during infection and sclerotial development compared with the vegetative growth stage. Further, study showed that *sop1* was essential for growth, sclerotial development and full virulence of *S. sclerotiorum*. *Sop1*-silenced transformants were more sensitive to high salt stress, fungicides and high osmotic stress. However, they were more tolerant to oxidative stress compared with the wild-type strain, suggesting that sop1 is involved in different stress responses and fungicide resistance, which plays a role in the environmental adaptability of *S. sclerotiorum*. Furthermore, a Delta blast search showed that microbial opsins are absent from the genomes of animals and most higher plants, indicating that sop1 is a potential drug target for disease control of *S. sclerotiorum*.

## Introduction

The white mold fungus *Sclerotinia sclerotiorum* (Lib.) de Bary is a typical necrotrophic phytopathogen with a remarkably broad host range and worldwide distribution. At least 408 described species of plants from 278 genera in 75 families are susceptible to this pathogen (Boland and Hall, 1994), and it often leads to great losses of economically important crops such as rapeseed (*Brassica* spp.), sunflowers, soybeans, and peanuts. There are several key steps in the life cycle of *S. sclerotiorum*: vegetative growth, infection, sclerotial development, sclerotial myceliogenic germination, sclerotial carpogenic germination, and apothecium formation (stipe). The sclerotia produced by *S. sclerotiorum* are pigmented, hard, asexual resting structures capable of surviving for years in soil (Adams and Ayers, 1979; Le Tourneau, 1979; Willetts and Bullock, 1992). Sclerotia can either germinate myceliogenically to produce mycelia or carpogenically to form apothecia, which can produce abundant ascospores. Ascospores can undergo airborne dissemination, which is the most important means of *S. sclerotiorum* dispersal (Chet and Henis, 1975; Abawi and Grogan, 1979; Steadman, 1979). Previous research has demonstrated that oxalic acid and a wide array of cell wall degrading enzymes were key pathogenic factors in this pathogen (Riou et al., 1991; Cessna et al., 2000) and that signal transduction pathways also played a crucial role in pathogenesis and sclerotial development (Chen et al., 2004; Erental et al., 2008).

Sensing and responding to the environment and ensuring appropriate cellular responses are vitally important for the survival and proliferation of many organisms in a range of biological niches. Organisms receive and respond to diverse extracellular signals or stimuli through cell signal-transduction cascades. Genes involved in the signal-transduction cascades are designated as signaling genes, and play subtle, versatile and crucial roles in diverse biological processes (Krauss, 2006). High osmolarity is a common extracellular signal that reflects the adaptation of organisms to various extreme environments. During pathogen-host interactions, an oxidative burst is a conserved and nonspecific defense reaction of plants against invading pathogens. In this process, reactive oxygen species (ROS) can be produced by both plants and pathogens around the infection site (Tiedemann, 1997; Schouten et al., 2002; Tenberge et al., 2002; Egan et al., 2007). Large quantities of ROS can kill pathogens, hence, oxidative stress is also a common extracellular stimuli that is crucial for the survival of pathogens during infection. Many signaling networks specifically respond to an increase in extracellular osmolarity, among which the Hog1-MAPK signaling pathway plays a particularly important role in cellular adaptation to osmotic stress (Brewster et al., 1993; Xu, 2000). In filamentous fungi, Hog1 homologs response for both osmoregulation and oxidative stress (Heller et al., 2012). In addition to Hog1 homologs, many other signaling genes are involved in the oxidative stress response, such as AP-1-like transcription factors that have been characterized as regulators of ROS resistance in mammalian cells as well as in fungi (Temme and Tudzynski, 2009).

Some signaling genes produce specialized integral membrane proteins known as receptors that initiate cell signal transduction and trigger cascade changes in cell functions (Seger and Krebs, 1995). Proteinaceous light sensors are a class of receptors generally grouped into three categories in plants: phototropins, cryptochromes, and phytochromes. These light sensors are actually chromoprotein complexes. Both phototropins and cryptochromes use flavin as the chromophore for blue-light or near-UV-light sensing, and the phytochromes use bilin as the chromophore for red-light or far-red-light sensing (Purschwitz et al., 2006). Similar photosensory systems have been found in fungi and have been confirmed to play multiple roles in fungal phototropism, photoconidiation, photomorphogenesis, circadian rhythm, pathogenicity, or virulence, light-dependent differentiation, secondary metabolism, pigmentation and asexual and sexual development (Idnurm and Heitman, 2005b; Lee et al., 2006; Liu and Bell-Pedersen, 2006; Purschwitz et al., 2006; Herrera-Estrella and Horwitz, 2007; Cai et al., 2013). In addition to the photosensory systems described above, the photosensory opsin system has been found in microbes and animals. Typical opsins are seven-transmembrane (TM) helix receptors that use retinal as a chromophore for light sensing (Luecke et al., 1999; Palczewski et al., 2000); thus, this opsin-retinal complex is referred to as rhodopsin (Zhang et al., 2011). A conserved lysine residue is located on TM helix seven (TM7), which is necessary for the incorporation of retinal within the 7-TM helices (Zhang et al., 2011). However, there are some opsin-like or opsin-related proteins (ORPs, such as some bacteriorhodopsins) that do not contain the retinal binding lysine and hence cannot function as opsins (Oesterhelt and Tittor, 1989; Brown, 2004). The opsins are divided into two distinct types: type I microbial opsins and type II animal opsins (Spudich et al., 2000). Although, the biological functions of many opsins in animals, prokaryotes and algae have been confirmed (Sineshchekov and Govorunova, 2001; Nickle and Robinson, 2007; Zhang et al., 2011), their roles in many fungi remains to be determined. Although, many opsins have been identified in fungal genomes, only a few fungal opsins, such as in *Neurospora crassa, Leptosphaeria maculans, Fusarium fujikuroi, Cryptococcus neoformans*, and *Botrytis cinerea*, have been documented, and they as yet have no known function (Bieszke et al., 1999; Idnurm and Howlett, 2001; Brown, 2004; Idnurm and Heitman, 2005a; Estrada and Avalos, 2009; Heller et al., 2012). The aim of this study was to elucidate the biological functions of a microbial opsin homolog gene, *sop1* in *S. sclerotiorum*. We found that *sop1* had diverse roles and was involved in growth, sclerotial development and virulence of *S. sclerotiorum*. *Sop1* was also involved in the osmotic stress and the oxidative stress responses and therefore partially contributed to the environmental adaptability of *S. sclerotiorum*.

## Materials and methods

### Fungal and bacterial strains, plants, and culture condition

*S. sclerotiorum* virulent wild-type strain Ep-1PNA367 (Xie et al., 2006) was used in this study. Fungal cultures were grown on potato dextrose agar (PDA, Difco, Detroit, MI, USA) at 20°C. *S. sclerotiorum* transformants were obtained and cultured on PDA amended with 80 μg/mL hygromycin B (EMD Biosciences, USA) at 20°C. *Escherichia coli* strain JM109 was used to propagate all plasmids. *Agrobacterium tumefaciens* strain EHA105 was used for fungal transformation. Seedlings of *Arabidopsis thaliana* (ecotype Columbia-0) were grown in greenhouse at 20 ± 2°C for 1 month under a 12 h light/dark cycle with 70% relative humidity.

### Construction of RNAi vectors and transformation of *S. sclerotiorum*

The vector pCXDPH with P*trpC*-*ccdB*-P*gpd* fragment (the promoter P*trpC* and P*gpd* were placed in opposite directions) and the vector pCIT with P*trpC*-intron-T*trpC* fragment (two same exogenetic gene fragments were inserted into this region in opposite directions to make the transcripts form “hairpin structure”) (Nguyen et al., 2008; Yu et al., 2012) were used to construct *S. sclerotiorum* RNAi vectors. In detail, a 530 bp *sop1* DNA fragment was amplified with a pair of specific primers RNAi-sop1-F (5′-CCATCGATGG ATCCCCAGTCCATCAGCCACTCCATCTGTT-3′) and RNAi-sop1-R (5′-CCCAAGCT TCTGCAGGAAAGCAACGCATCCCA TAGCATACCAA-3′) from the cDNA library of *S. sclerotiorum* and then ligated into pCXDPH digested by *Xcm*I (New England Biolabs, Beverly, MA, USA) to replace the *ccdB* gene to produce the pRNAi-1 vector (**Figure 3A**). The same amplified *sop1* DNA fragment was digested with two pairs of suitable enzymes, respectively, and then both of the digested DNA fragments were ligated into the pCIT vector. Afterwards, the fusion fragment P*trpC*-*sop1* fragment-intron-*sop1* fragment-T*trpC* was digested with *Sac* I and *Xho* I before it was ligated into the pCH vector (Yu et al., 2012) to produce the pRNAi-2 vector (Figure S2A). The *Agrobacterium*-mediated transformation (ATMT) with mycelial agar discs method was used to transform *S. sclerotiorum* as previously described (Yu et al., 2012). The knockdown transformants were validated by quantitative reverse transcription PCR (qRT-PCR).

### Extraction and manipulation of nucleic acids

To examine the *sop1* expression patterns during infection, fresh hyphae of the wild-type strain without any medium were inoculated on *A. thaliana* (Col-0) leaves and cultured at 20°C. The hyphae and inoculated leaves were collected together at 0, 3, 6, 9, and 12 h post inoculation (hpi), respectively, for total RNA extraction. To examine the *sop1* expression patterns during sclerotial development, mycelial agar discs of the wild-type strain were taken from active colony edge and inoculated on the center of cellophane over PDA plates, and cultured at 20°C for 1–7 days post inoculation (dpi) before the cultures were collected, respectively, for total RNA extraction. This period was selected because it represents the entire biological processes from vegetative growth, the initial stages of sclerotial development to the sclerotial formation and maturation stages. To evaluate the relative *sop1* expression levels in the wild-type strain and different transformants, mycelial agar discs were taken from active colony edge and inoculated on the center of cellophane over PDA plates, and cultured at 20°C for 7 dpi and then the cultures (including both mycelium and sclerotia) were collected for total RNA extraction. To examine the *sop1* expression patterns when the wild-type strain were cultured with oxidative stress, the mycelial agar discs were taken from active colony edge and inoculated on the center of cellophane over complete medium (CM) plates without any treatment and CM plates supplemented with different concentrations of H_2_O_2_ (0–12 mM), respectively, and cultured at 20°C for 24 h before the cultures were collected for total RNA extraction. The total RNA was isolated from each sample described above with TriZOL reagent (Invitrogen, USA) according to the manufacturer's protocols. First strand cDNA was generated with RevertAid™ First Strand cDNA Synthesis Kit (MBI Fermentas, Lithuania) after the total RNA samples were treated with DNase I (TaKaRa, Dalian). Gene expression was analyzed by qRT-PCR using a Bio-Rad CFX96 Real Time System (America) and Quantitect SYBR Green PCR master mix (Bio-Rad, USA), according to the manufacturer's instructions. The *S. sclerotiorum* β-tubulin gene (SS1G_04652) was used as housekeeping gene for qRT-PCR normalization (Harel et al., 2006). For qRT-PCR analysis, the primers qPCR-sop1-F (5′-TTCCAAGTGTTGTTCCT AATGC-3′) and qPCR-sop1-R (5′-GAGAGTGATGAATGCGGTAATAAC-3′) were used to amplify the *sop1* gene fragment; the primers β-tubulin-F (5′-TTGGATTTGCTCC TTTGACCAG-3′) and β-tubulin-R (5′-AGCGGCCATCATGTTCTTAGG-3′) were used to amplify the β-tubulin gene fragment. For every experiment, three samples were harvested and independent qRT-PCR assays were repeated three times, with each repetition having three replicates.

### Characterization of *Sop1* silenced transformants

To examine colony morphology and assay growth rates, mycelial agar discs (0.8 mm) of the silenced transformants and the wild-type strain were taken from active colony edge and inoculated on the center of PDA Petri dishes, and cultured at 20°C. Colony diameters were measured at 24 hpi and the first day growth rate (semidiameter of the colony minus semidiameter of the mycelial agar disc) was calculated for statistical analysis. To compare the morphology of hyphal tips, mycelial agar discs of the silenced transformants and the wild-type strain were taken from active colony edge and inoculated on cellophane over PDA plates, and cultured for 48 h at 20°C before the morphology of hyphal tips were examined using a light microscope. To evaluate virulence, mycelial agar discs (diameter 6 mm) of the silenced transformants and the wild-type strain were taken from active colony edge and inoculated on detached *Brassica napus* and tomato leaves, and cultured at 20°C for 48 h before the induced lesions were measured. To calculate the inhibition rate of hyphal growth when the silenced transformants and the wild-type strain were cultured with high osmotic stress, mycelial agar discs (diameter 8 mm) were taken from active colony edge and inoculated on the center of CM plates without any treatment and CM plates supplemented with 1 M glucose, 1 M sorbitol and 1.2 M sucrose, respectively, and cultured for 24 h at 20°C before the colony diameters were measured. To measure the colony diameters of the silenced transformants and the wild-type strain cultured with membrane damage stress, high salt stress, fungicide stress, and oxidative stress, the mycelial agar discs (diameter 8 mm) of the silenced transformants and the wild-type strain were taken from active colony edge and inoculated on the center of CM plates without any treatment and CM plates supplemented with 0.02% sodium dodecylsulphate (SDS), 1 M NaCl, 1 M KCl, 0.25 μg/mL carbendazim, 0.40 μg/mL dimetachlone, 0–24 mM H_2_O_2_, respectively, and cultured at 20°C. Colony diameters were measured every day before the colony reached margins of the plates. In all experiments, at least three independent replications were performed.

### Bioinformatics analysis

The signaling genes were identified according to the genome annotation of *S. sclerotiorum* (Amselem et al., 2011). The Phyre2 server (Kelley and Sternberg, 2009) was used to predict transmembrane domain regions and construct 3D structural model which was displayed by PyMOL (DeLano, 2002). Sequences for sop1 homologs were collected from the non-redundant protein sequence database at the NCBI website^1^ using DELTA-BLAST (Boratyn et al., 2012) with sop1 as the query sequence. Datasets were assembled with a cut-off *E*-value < 1e-6. All sequences available for sop1 homologs form *Volvox carteri* f. nagariensis, *Dunaliella salina, Acetabularia acetabulum, Chlorella vulgaris, Guillardia theta* CCMP2712, *Podospora anserina* S mat+, *L. maculans* JN3, *Haloarcula argentinensis, Halobacterium salinarum, Natronomonas pharaonis, Haloarcula marismortui, Chlamydomonas reinhardtii, Cryptomonas* sp. S2, *Cyanophora paradoxa, N. crassa* OR74A, *F. fujikuroi, C. neoformans* var. grubii H99, *C. neoformans* var. neoformans JEC21, *B. cinerea* B05.10 and *Oryza sativa* Indica Group were used to perform amino acid alignment using COBALT (Papadopoulos and Agarwala, 2007). These species were selected because their opsin genes were experimentally characterized or reviewed previously (Bieszke et al., 1999; Idnurm and Howlett, 2001; Brown, 2004; Ruiz-Gonzalez and Marin, 2004; Idnurm and Heitman, 2005a; Estrada and Avalos, 2009; Zhang et al., 2011; Heller et al., 2012). Multiple protein sequence alignment without any truncation was viewed and edited by Jalview (Clamp et al., 2004). The entire protein sequence alignment was subjected to model testing with the ProtTest v.2.4, using the Aikake information criterion (AIC) and default settings (Abascal et al., 2005), before it was used for building a phylogenetic tree. Protein maximum likelihood (ML) tree was inferred with PhyML-mixtures (Guindon and Gascuel, 2003; Le et al., 2008), assuming the best-fit LG model (Le and Gascuel, 2008) according to the ProtTest and SPR tree topology search strategy (Hordijk and Gascuel, 2005). Gaps in alignment were systematically treated as unknown characters. The reliability of internal branches was evaluated based on aLRT SH-like branch support (Anisimova and Gascuel, 2006). The phylogenetic tree graphic was produced using the Interactive Tree of Life (Letunic and Bork, 2007).

## Results

### Characterization of the microbial opsins in *S. sclerotiorum* genome

**Figure 1:**
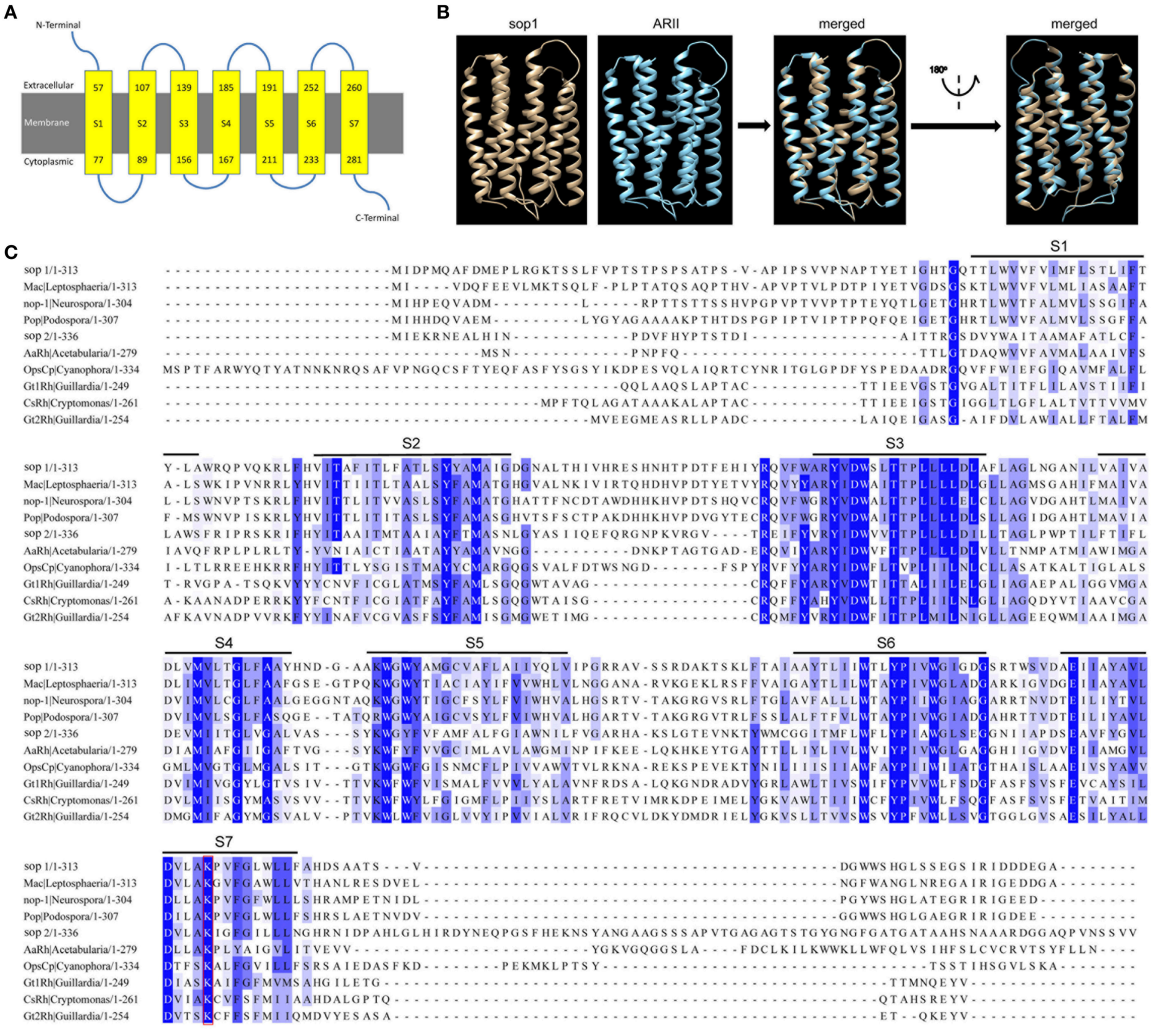
**Characterization of sop1 in ***S. sclerotiorum*****. **(A)** A schematic diagram of seven transmembrane structures (S1–S7, yellow boxes) that were predicted with the Phyre2 program. Numbers in the yellow boxes indicate start and end sites of each transmembrane helix of sop1, respectively. **(B)** Comparison of 3D structural models of sop1 (from 50 aa to 287 aa) and eukaryotic light-driven proton pumping rhodopsin, Acetabularia rhodopsin II (arII), from marine alga *A. acetabulum* (Wada et al., 2011). The 3D structural models were generated with the Phyre2 program. 218 residues (70% of sop1) have been modeled with 100% confidence by the single highest scoring template c3am6C in the Phyre 2 database. **(C)** Multiple alignment of the protein sequences of sop1, sop2, Mac in *L. maculans* (GenBank accession: AAG01180), nop-1 in *N. crassa* OR74A (GenBank accession: XP_959421), Pop in *P. anserine* S mat+ (GenBank accession: XP_001904282), AaRh in *A. acetabulum* (GenBank accession: AAY82897), OpsCp in *C. paradoxa* (GenBank accession: ACV05065), Gt1Rh in *G. theta* (GenBank accession: ABA08437), CsRh in *Cryptomonas* sp. S2 (GenBank accession: ABA08439), and Gt2Rh in *G. theta* (GenBank accession: ABA08438) using COBALT program.

Our previous study on the transcriptomic analysis of the six key developmental stages (including vegetative growth, infection, sclerotial development, sclerotial myceliogenic germination, sclerotial carpogenic germination, and apothecium formation) of *S. sclerotiorum* based on digital gene expression (DGE) (Lyu et al., 2015) revealed the expression of a large number of signaling genes that were significantly regulated during corresponding biological processes, as summarized in Table S1. A microbial opsin homolog gene (SS1G_01614) is very special among these signaling genes because it was dramatically induced during all developmental stages compared with the vegetative growth stage (Table S1) and therefore was selected for further study. Two microbial opsin homologs were found in *S. sclerotiorum* genome: SS1G_01614 (GenBank accession: XP_001597420) and SS1G_04339 (GenBank accession: XP_001594532) and these were designated as sop1 and sop2, respectively, in this study. Sop1 and sop2 have high protein sequence similarity (*E*-value: 3e–33). Sop1 consists of 313 amino acid residues with a bacteriorhodopsin domain (pfam01036, *E*-value: 1.93e–25). It has seven predicted transmembrane helices and its initial N terminus and C terminus were predicted to be extracellular and cytoplasmic, respectively (Figure 1A). The predicted three dimensional (3D) structure of sop1 showed that it shared a strong structural similarity with the eukaryotic light-driven proton-pumping rhodopsin *Acetabularia* rhodopsin II (ARII) from the marine alga *A. acetabulum* (Wada et al., 2011; Figure 1B). The multiple protein sequence alignment of sop1 and sop2 with Mac in *L. maculans* (Idnurm and Howlett, 2001), nop1 in *N. crassa* OR74A (Bieszke et al., 1999), Pop in *Podospora anserine* S mat+ (Espagne et al., 2008), AaRh in *A. acetabulum* (Tsunoda et al., 2006), OpsCp in *C. paradoxa* (Frassanito et al., 2010), Gt1Rh in *G. theta* (Sineshchekov et al., 2005), CsRh in *Cryptomonas* sp. S2 (Sineshchekov et al., 2005), and Gt2Rh in *G. theta* (Sineshchekov et al., 2005) indicated that the lysine residues of these microbial opsins in the last transmembrane domains were conserved (Figure 1C). In addition, it also showed that the seven TM regions and their contiguous regions in these proteins were more conserved than other regions (Figure 1C).

### Expression patterns of *Sop1*

**Figure 2:**
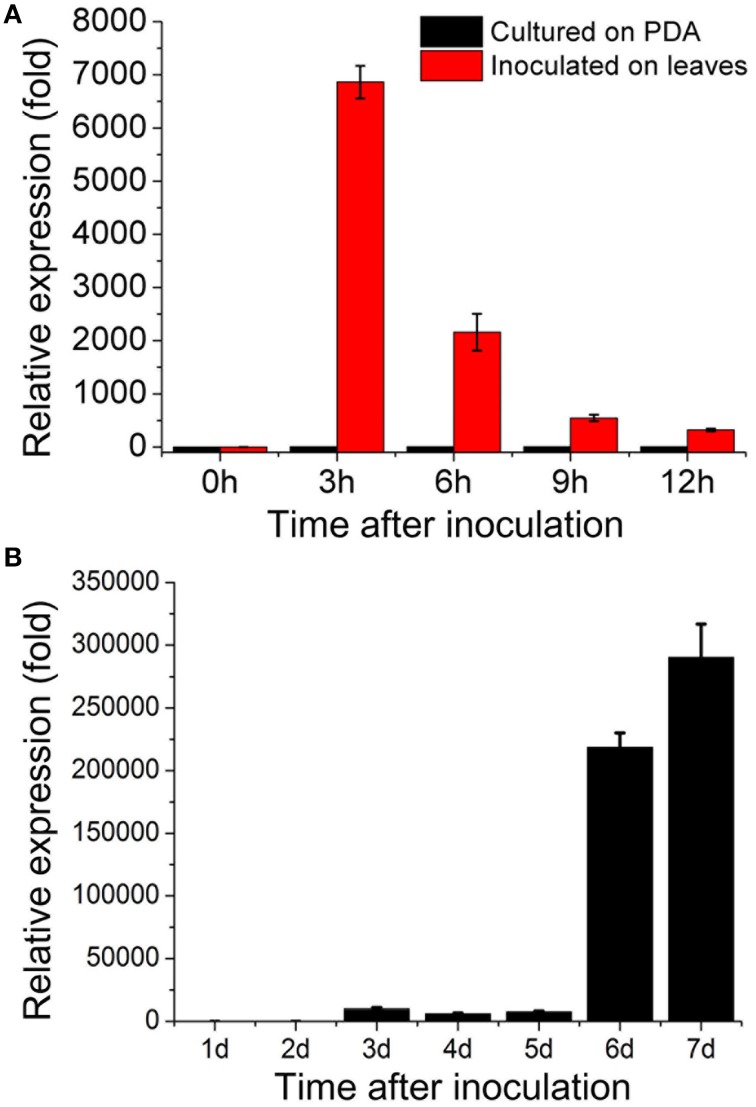
**Expression patterns of ***sop1*****. **(A)** Relative expression of *sop1* during infection and vegetative growth as determined by qRT-PCR analysis. The abundance of cDNA from cultures on PDA at 0 hpi was assigned a value of 1. **(B)** Relative expression of *sop1* during sclerotial development as determined by qRT-PCR analysis. The abundance of cDNA from cultures on PDA at 1 dpi was assigned a value of 1. The gene expression levels of *sop1* in the wild-type strain were normalized to that of the β-tubulin transcripts from each sample. Bars indicate the standard error.

Our DGE data indicated that *sop1* was not expressed during the vegetative growth stage, but was dramatically up-regulated above 10-fold (log_2_Ratio) during all other developmental stages (Table S1). To examine the expression patterns of *sop1* during infection and sclerotial developmental in more detail, qRT-PCR was used to detect *sop1* expression levels at different times after inoculation. The results showed that *sop1* was dramatically up-regulated during the very early stage of infection in *A. thaliana* (Col-0) leaves. The expression level of *sop1* reached a peak at 3 hpi before it gradually decreased during the later stages of infection (6, 9, and 12 hpi, Figure 2A). In addition, the qRT-PCR results showed that the expression of *sop1* was dramatically induced during the initial stage of sclerotial development (at 3 dpi) and up-regulated approximately 300,000-fold during the sclerotial maturation stage (at 7 dpi, Figure 2B). Taken together, these results were in accordance with the DGE data and suggested that *sop1* was possibly involved in the infection and sclerotial developmental processes of *S. sclerotiorum*.

### RNA interference-mediated down-regulation of *Sop1* impaired growth, sclerotial development, and virulence of *S. sclerotiorum*

**Figure 3:**
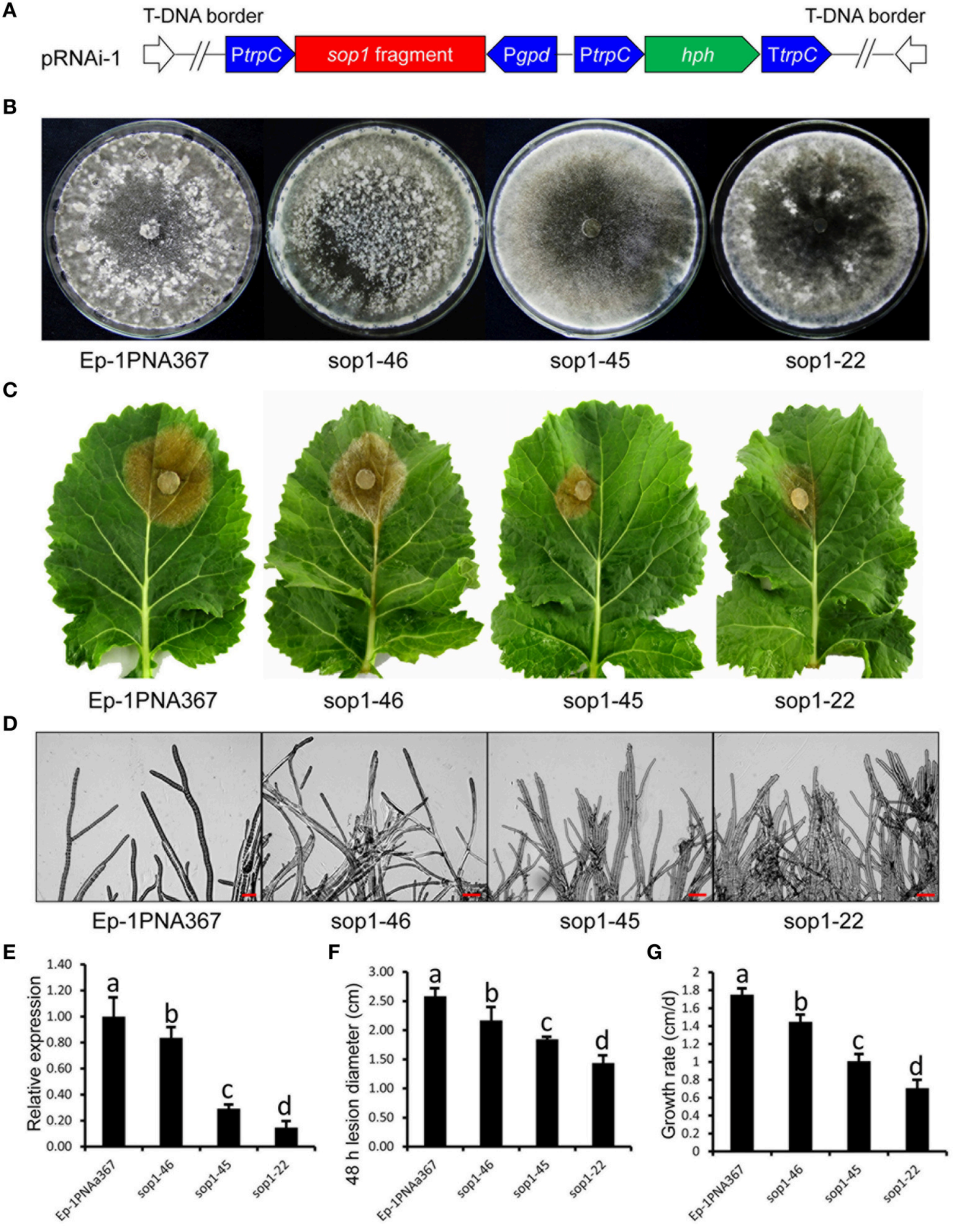
**Biological characterization of the ***sop1*** silenced transformants**. **(A)** Construction of *sop1* silencing vector pRNAi-1. **(B)** Comparison of the phenotypes of the *sop1* silenced transformants and the wild-type strain. Photos were taken at 8 dpi. **(C)** Virulence assay of the silenced transformants and the wild-type strain on detached oilseed rape leaves. **(D)** Morphological comparison of the hyphal tips of the *sop1* silenced transformants and the wild-type strain. **(E)** Relative *sop1* expression level of the *sop1* silenced transformants and the wild-type strain as determined by qRT-PCR analysis. A value of 1 was assigned to the abundance of cDNA from the wild-type strain. The gene expression levels of *sop1* in the silenced transformants and the wild-type strain were normalized to that of the β-tubulin transcripts from each strain. Bars indicate the standard error. **(F)** Comparison of lesion diameter for the silenced transformants and the wild-type strain. **(G)** Comparison of the hyphal growth rate of the silenced transformants and the wild-type strain. Three independent replications were performed. Bars indicate the standard error. The values are presented as the mean ± s.d. Differentiation was evaluated by a *t*-test. Different letters on a graph indicate significant differences, while same letters on a graph indicate no significant differences, *P* = 0.05.

Because of multi-nucleated cells of *S. sclerotiorum*, the RNAi technique was used to study the biological functions of *sop1*. We had noted the “off-target” effect of the RNAi technique before silencing *sop1*. Although the protein sequence similarity between sop1 and sop2 is high, there was no sequence similarity between *sop1* transcript and all other *S. sclerotiorum* transcripts, even between the transcripts of *sop1* and *sop2* according to a BLASTN search. Additionally, *sop2* is almost not expressed during vegetative growth, sclerotial development and infection as well as all the other developmental stages according to our DGE data (Lyu et al., 2015). Two strategies (Figure 3A and Figure S2A) were used to silence the *sop1* gene in the wild-type strain Ep-1PNA367. QRT-PCR analysis was used to examine the accumulation of *sop1* transcripts in each transformant. The results showed that the silenced transformants with markedly reduced expression levels of *sop1* exhibited abnormal sclerotial development and significantly reduced virulence. For example, the silenced transformants sop1-22 and sop1-45 showed abnormal sclerotial development when they were cultured on PDA at 20°C for 8 days, while the wild-type strain had already produced large amount of mature sclerotia under the same conditions (Figure 3B). Ten days later, sop1-22 and sop1-45 produced a small amount of immature sclerotia indicating that the sclerotial development in sop1-22 and sop1-45 was severely delayed. A similar result was obtained by the other silencing strategy (Figure S2B). Compared with the wild-type strain, the tip hyphae of sop1-22 and sop1-45 were denser and more likely to adhere to each other (Figure 3D), and the hyphal growth rate was also significantly reduced in sop1-22 and sop1-45 (Figure 3G and Figure S2F). The virulence of sop1-22 and sop1-45 were also significantly impaired (Figures 3C,F). Similar results were observed when sop1-22 and sop1-45 were inoculated on detached tomato leaves (Figure S1), indicating that the virulence reduction was not species-specific. Similar results were observed for the other silencing strategy (Figures S2C,E). In addition, the silencing efficiency was positively correlated with the virulence reduction (Figures 3E,F; Figures S1, S2D,E); for example, the lesions caused by another transformant (sop1-46) with lower silencing efficiency were larger than those caused by sop1-22 with higher silencing efficiency. This phenomenon indicated that the phenotypic change of silenced transformants was caused by the silencing of *sop1*. Taken together, our results showed that *sop1* has pleiotropic effects on sclerotial development, virulence and hyphal growth of *S. sclerotiorum*.

### *Sop1* is involved in the adaptability of *S. sclerotiorum* to different environmental stressors

**Figure 4:**
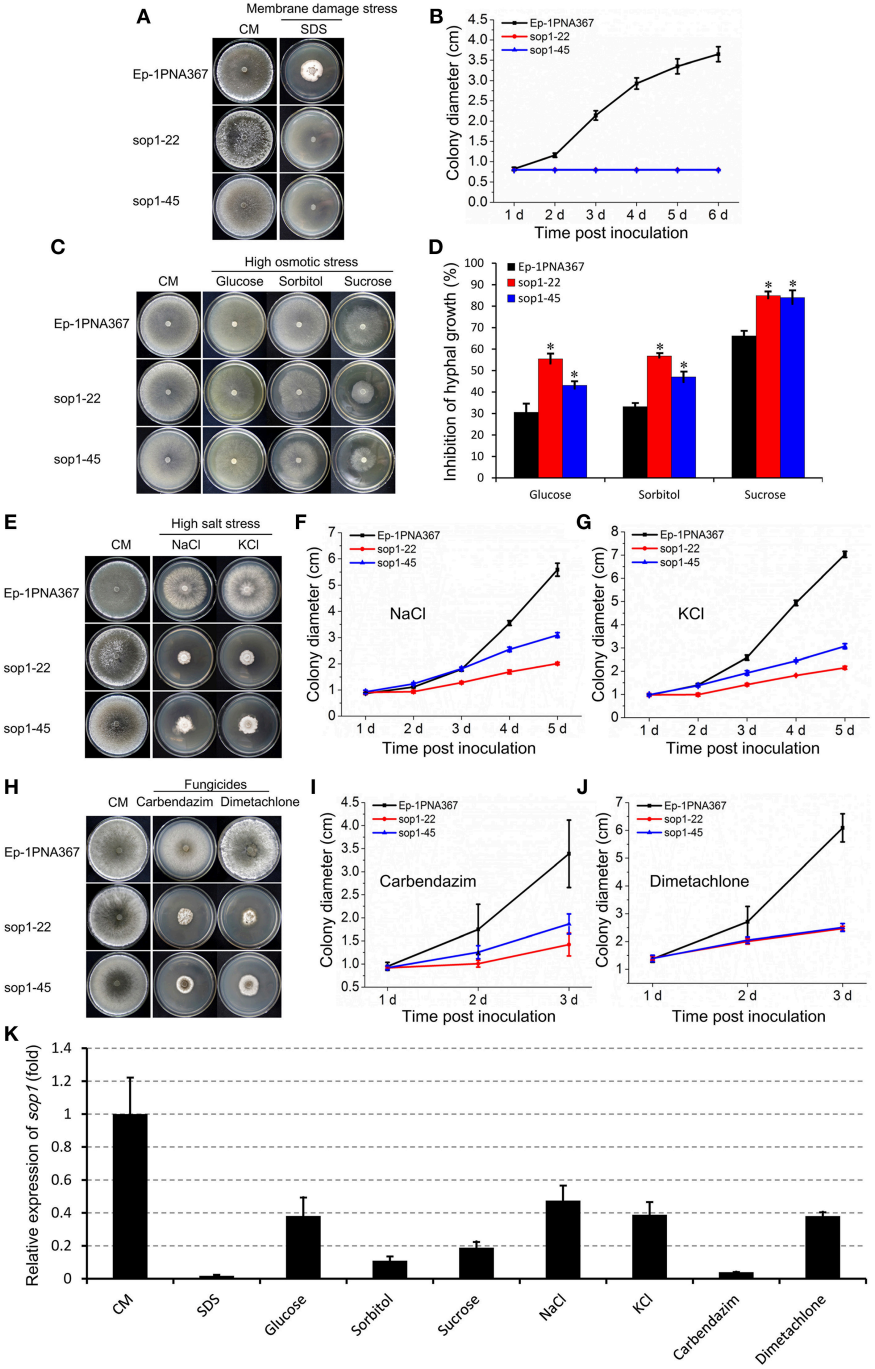
**Growth of ***sop1*** silenced transformants in the presence of various stressors**. **(A)** Phenotypes of the *sop1* RNAi-silenced transformants and the wild-type strain grown on CM supplemented with 0.02% SDS at 20°C. Photos were taken at 6 dpi. **(B)** The colony diameter of the *sop1* RNAi-silenced transformants and the wild-type strain on CM supplemented with 0.02% SDS at different times. **(C)** Phenotypes of the *sop1* RNAi-silenced transformants and the wild-type strain that grown on CM supplemented with glucose (1 M), sorbitol (1 M), and sucrose (1.2 M), respectively, at 20°C. Photos were taken at 3 dpi. **(D)** The inhibition rate of the hyphal growth of the *sop1* RNAi-silenced transformants and the wild-type strain on stress-inducing media. ^*^ on the graph indicate significant differences, *P* = 0.05. **(E)** Phenotypes of the *sop1* RNAi-silenced transformants and the wild-type strain grown on CM supplemented with NaCl (1 M) and KCl (1 M), respectively, at 20°C. Photos were taken at 6 dpi. **(F,G)** The colony diameter of the *sop1* RNAi-silenced transformants and the wild-type strain on CM supplemented with 1 M NaCl and 1 M KCl, respectively, at different times. **(H)** Phenotypes of the *sop1* RNAi-silenced transformants and the wild-type strain grown on CM supplemented with carbendazim (0.25 μg/mL) and dimetachlone (0.40 μg/mL), respectively, at 20°C. Photos were taken at 6 dpi. **(I,J)** The colony diameter of the *sop1* RNAi-silenced transformants and the wild-type strain on CM supplemented with 0.25 μg/mL carbendazim and 0.40 μg/mL dimetachlone, respectively, at different times. **(K)** Relative expression of *sop1* in the wild-type strain in the various stress conditions tested as determined by qRT-PCR analysis. Samples were collected for gene expression detection at 3 dpi on CM without any treatment and CM with 0.02% SDS, 1 M glucose, 1 M sorbitol, 1.2 M sucrose, 1 M NaCl, 1 M KCl, 0.25 μg/mL carbendazim, and 0.40 μg/mL dimetachlone, respectively. The abundance of cDNA from cultures on CM without any treatment was assigned a value of 1. The gene expression levels of *sop1* were normalized to that of the β-tubulin transcripts from each sample. In all experiments at least three independent replications were performed, and the values are presented as the mean ± s.d. Bars indicate the standard error.

Because sop1 was predicted to be a transmembrane protein, the tolerance of *sop1* silenced transformants to membrane damage stress was tested. In the presence of 0.02% SDS, which attacks the cell membrane (Temme et al., 2012), the growth rate of both the silenced transformants (sop1-22 and sop1-45) and the wild-type strain was dramatically suppressed during the entire growth process on CM. However, the wild-type strain could grow slowly at the later stages after inoculation on CM with SDS, while the growth of silenced transformants sop1-22 and sop1-45 was completely inhibited on CM with SDS even during the later stages after inoculation (Figures 4A,B), suggesting that the *sop1* silenced transformants were more sensitive to SDS. This result indicated that *sop1* might be involved in the tolerance to the cytomembrane damage stress, or *sop1* might affect the homeostasis of *S. sclerotiorum*. To further explore the biological roles of *sop1* under different environmental stresses, *sop1* silenced transformants and the wild-type strain were cultured with high concentrations of sugars and salts. When the *sop1* silenced transformants and the wild-type strain were cultivated with a high concentration of glucose (1 M), sorbitol (1 M), and sucrose (1.2 M), the growth of sop1-22 and sop1-45 was more significantly inhibited compared with the wild-type (Figures 4C,D), suggesting that the silencing of *sop1* made *S. sclerotiorum* more sensitive to high osmotic stress. When *sop1* silenced transformants and the wild-type strain were cultivated with a high concentration of salts, the growth rate of sop1-22, sop1-45 and the wild-type strain were all severely decreased during the initial stages (1-2 dpi, Figures 4F,G). For example, the colony diameter of untreated sop1-22, sop1-45, and the wild-type strain were 2.63 ± 0.12, 2.81 ± 0.10, and 3.52 ± 0.14 cm, respectively, at 1 dpi on CM without any treatment; but the colony diameter of sop1-22, sop1-45 and the wild-type strain were only 0.91 ± 0.08, 0.93 ± 0.07, and 0.87 ± 0.04 cm, respectively, at 1 dpi on CM with 1 M NaCl; and were only 0.98 ± 0.04, 0.98 ± 0.06, and 0.97 ± 0.03 cm, respectively, at 1 dpi on CM with 1 M KCl. However, the wild-type strain grew better than sop1-22 and sop1-45 after 4 dpi on CM with 1 M NaCl and 1 M KCl (Figures 4E–G). For example, the colony diameter of the wild-type strain was 5.59 ± 0.25 cm and 7.03 ± 0.13 cm at 5 dpi on CM with 1 M NaCl and 1 M KCl, respectively. But the colony diameter of sop1-22 and sop1-45 were only 2.01 ± 0.05 cm and 3.10 ± 0.09 cm, respectively, on CM with 1 M NaCl, and were only 2.15 ± 0.07 cm and 3.07 ± 0.11 cm, respectively, on CM with 1 M KCl. These results indicated that although the growth of both of the *sop1* silenced transformants and the wild-type strain was dramatically depressed during the initial stages, the capacity to overcome high salt stress of the wild-type strain was greater than that of the *sop1* silenced transformants, and therefore the silenced transformants were more sensitive to high salt stress during the later stages. Likewise, similar results were obtained when the silenced transformants and the wild-type strain were inoculated on CM supplemented with the two fungicides carbendazim and dimetachlone, respectively. The results showed that although the growth of all strains was severely depressed during the initial stage (1 dpi, Figures 4I,J), the *sop1* silenced transformants sop1-22 and sop1-45 were more sensitive to the two fungicides than the wild-type during the later stages (3 dpi, Figures 4H–J). Additionally, our qRT-PCR result showed that the expression of *sop1* was significantly down-regulated in the various stress conditions (Figure 4K). Taken together, these results indicated that *sop1* was involved in the response to high osmotic stress, high salt stress and fungicides resistance, and might partially contribute to the capacity of *S. sclerotiorum* to overcome extreme environmental factors. Thus, *sop1* is associated with the environmental adaptability of *S. sclerotiorum*.

### *Sop1* is involved in the tolerance of *S. sclerotiorum* to oxidative stress

**Figure 5:**
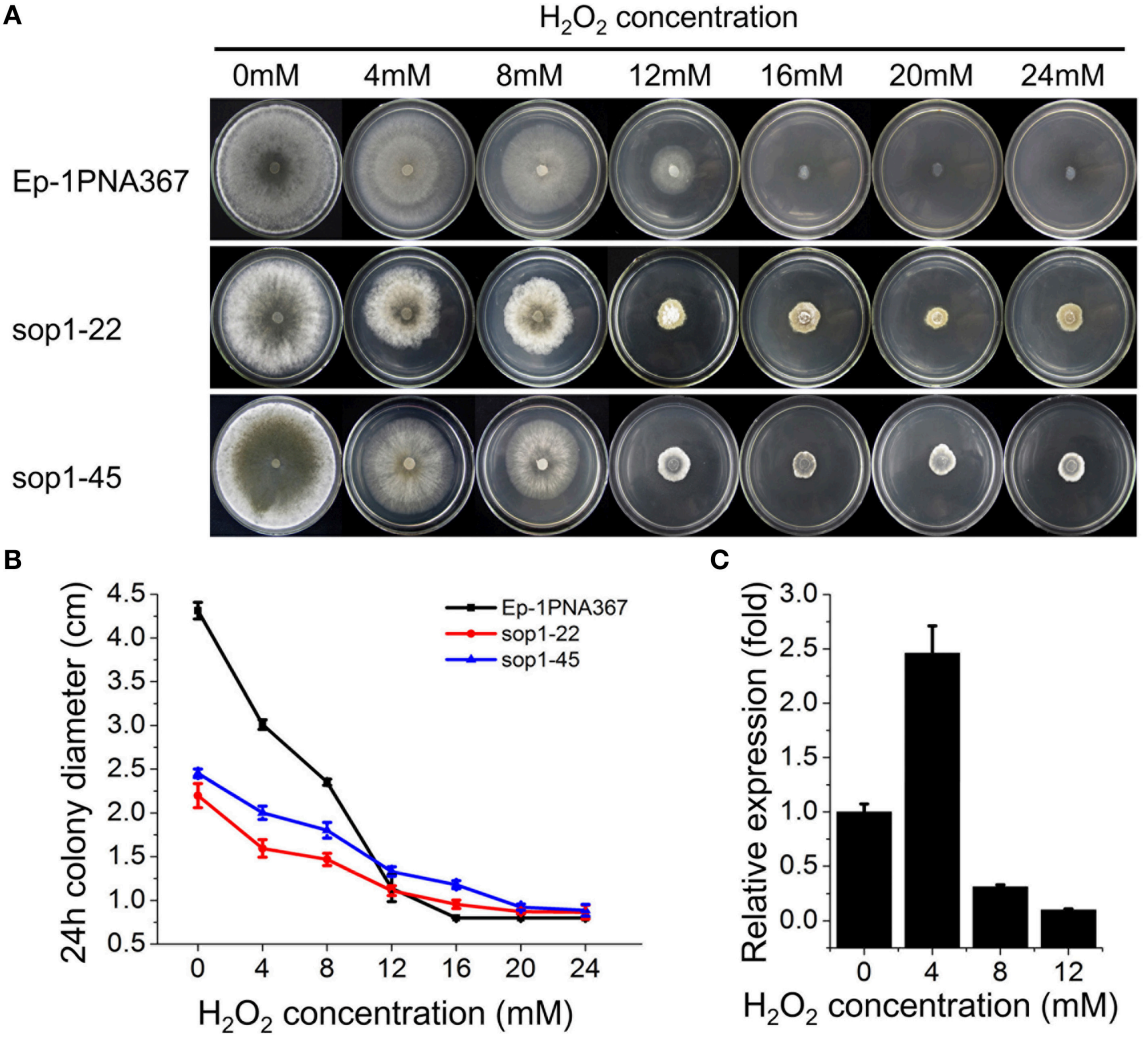
**Tolerance of ***sop1*** silenced transformants to oxidative stress. (A)** Phenotypes of the *sop1* silenced transformants and the wild-type strain on CM supplemented with different concentrations of H_2_O_2_. Photos were taken at 5 dpi. **(B)** The colony diameter of the *sop1* silenced transformants and the wild-type strain on CM supplemented with different concentrations of H_2_O_2_ at 24 hpi. **(C)** The *sop1* relative expression levels when the wild-type strain was inoculated on CM with different concentrations of H_2_O_2_ as indicated by qRT-PCR analysis. The abundance of cDNA from cultures on CM without H_2_O_2_ was assigned a value of 1. The gene expression levels of *sop1* were normalized to that of the β-tubulin transcripts from each sample. In all experiments three independent replications were performed, and the values are presented as the mean ± s.d. Bars indicate the standard error.

Previous studies showed that the expression of *bop1* (a *sop1* homologous gene) in *B. cinerea*, a necrotrophic fungus with a close phylogenetic relationship to *S. sclerotiorum*, were induced upon H_2_O_2_ exposure (Temme and Tudzynski, 2009; Heller et al., 2012; Temme et al., 2012), suggesting that the opsins of filamentous fungi are involved in the oxidative stress response. To explore the role of *sop1* in the oxidative stress response, the *sop1* silenced transformants and the wild-type strain were cultured on CM supplemented with different concentrations of H_2_O_2_. Intriguingly, our results showed that the degree of the growth inhibition of the wild-type stain on CM supplemented with H_2_O_2_ was obviously greater than that of sop1-22 and sop1-45, indicating that silencing *sop1* made *S. sclerotiorum* more tolerant to higher concentrations of H_2_O_2_ (Figures 5A,B). The qRT-PCR results showed that the expression of *sop1* was induced when the wild-type strain was inoculated on CM with a low concentration of H_2_O_2_ (4 mM), however, it was down-regulated with a high concentration of H_2_O_2_ (8 and 12 mM, Figure 5C). This result indicated that *S. sclerotiorum* may adapt to oxidative stress partially through the regulation of *sop1* expression, which is in accordance with a previous result (Temme and Tudzynski, 2009). Taken together, these results indicated that *sop1* was involved in the response to oxidative stress, and it might play many roles in the response to various environmental signals.

### A phylogenetic analysis of microbial opsins

**Figure 6:**
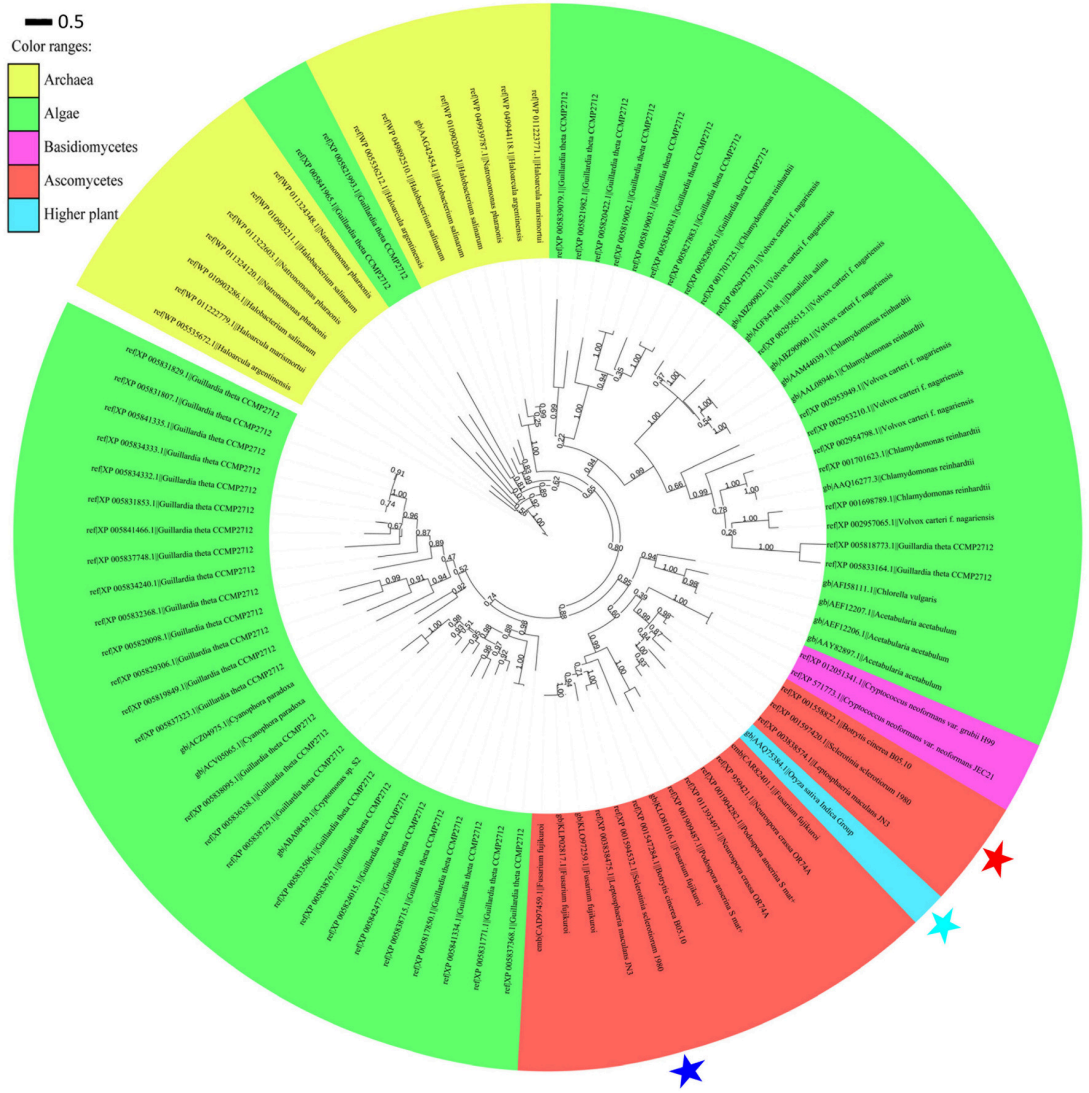
**Phylogenic analysis of type I microbial opsin homologs**. A phylogenetic tree was built using the PhyML-mixtures based on a multiple protein sequence alignment generated using COBALT with the Constraint *E*-value parameter setting to 0.003. The approximate likelihood ratios (SH-test) are indicated. Sop1, sop2, and the putative rhodopsin (GenBank accession: AAQ75384.1) in the higher plant *O. sativa* indica group are indicated by the red, blue, and sky blue stars, respectively. The characters before and after “||” indicate the GenBank accession numbers and corresponding species and strains, respectively. The scale bar corresponds to 0.5 amino acid substitutions per site.

An exhaustive search using DELTA-BLAST in the non-redundant protein sequence database at NCBI for sop1 showed that sop1 homologs were widespread in archaea, ascomycetes, basidiomycetes and lower plants such as algae (data not shown here), and the only exception was the presence of a putative rhodopsin (GenBank accession: AAQ75384.1) in the higher plant *O. sativa* indica group (Figure 6, see below), which was considered to be a horizontal gene transfer (HGT) event (Ruiz-Gonzalez and Marin, 2004). Additionally, not all sequenced species of bacteria, fungi or lower plants contain microbial opsins. This patchy distribution was thought to be caused by gene losses in multiple lineages in evolution (Ruiz-Gonzalez and Marin, 2004). All homology sequences of sop1 derived from the selected organisms (described in Materials and Methods) and *S. sclerotiorum* were used to perform a phylogenetic analysis, which showed that the microbial opsins had different copy numbers in different species (Figure 6). For example, there were 38 copies of microbial opsin homologs in *G. theta*, while most ascomycetes only had one or two copies (Figure 6). This phenomenon indicated that gene duplication events in microbial opsins occurred in some organisms in evolution. For the microbial opsins that have been experimentally studied or reviewed, sop1 appeared in the same branch with proton pumps: AaRh in *A. acetabulum*, Pop in *P. Anserine* S mat+ and Mac in *L. maculans* (Zhang et al., 2011), and had relatively distant phylogenetic relationship with the histidine kinase rhodopsin VcCop6 (GenBank accession: XP_002957065) in the multicellular green alga *V. carteri* f. nagariensis (Figure 6; Prochnik et al., 2010). Thus, the function of sop1 may have a closer phylogenetic relationship with the microbial proton-extruding pumps, that is accordance with the 3D structural analysis of sop1 (described as above).

## Discussion

Many signaling genes were significantly regulated not only during infection but also during multiple developmental stages, indicating that these genes may play diverse roles during the corresponding biological processes and that defective mutants of these genes may have a pleiotropic phenotype. Previous research demonstrated that the signaling gene-related, pathogenicity-defective strains of *S. sclerotiorum* often exhibit abnormal sclerotial development (Rollins, 2003; Erental et al., 2007; Jurick and Rollins, 2007). In this study, the *sop1* gene was also experimentally confirmed to be involved in growth, sclerotial development and virulence of *S. sclerotiorum* in accordance with its expression patterns (Table S1). These results indicated *sop1* affected the basal development of *S. sclerotiorum*, and therefore indirectly affected its sclerotial development and virulence.

Undoubtedly, light is very important for the regulation of various physiological processes for almost all life on Earth. The type I opsins are photoreceptors that harvest light energy to carry out metabolic processes. Although this family also includes some bacteriorhodopsin-like proteins that do not contain the retinal binding lysine and cannot function as opsins (Oesterhelt and Tittor, 1989), sop1 is obviously not grouped with these proteins because it has a conserved lysine residue in the last transmembrane domains. *Sop1* was found to be necessary for full virulence and sclerotial development of *S. sclerotiorum*. However, unlike the crucial roles of light-dependent regulation during sclerotial development and virulence in other fungi (Calvo et al., 2004; Duran et al., 2007; Canessa et al., 2013), the role of light-dependent regulation in sclerotial development and virulence seems not to be significant in *S. sclerotiorum* because our experience indicated no obvious differences when this fungus was cultured in the dark or in the light under normal laboratory conditions during sclerotial development and infection. This result indicated that the roles of sop1 in sclerotial development and infection and the inferred traditional molecular function of sop1 (light-driven proton-pumping) are independent because these biological functions of sop1 are independent on light while the molecular function of sop1 relies on light.

The expression patterns of *sop1* indicated that the expression of *sop1* has a intimate relationship with the development of *S. sclerotiorum* because the expression level of *sop1* was very low during vegetative growth stage while the expression of *sop1* was significantly up-regulated during later stages on PDA, especially during the mature period of sclerotial formation. Previous research showed that *bop1* expression was induced when *B. cinerea* hyphae were treated with 10 mM H_2_O_2_ for 30 min (Temme and Tudzynski, 2009; Heller et al., 2012; Temme et al., 2012), indicating that the expression of opsin genes in filamentous fungi is regulated by oxidative stress. Our qRT-PCR and DGE data further showed that the expression of *sop1* was regulated not only by oxidative stress but also by the developmental cycle itself because it was dramatically up-regulated during all developmental stages compared with the vegetative growth stage. However, our results indicated that *sop1* expression was reduced when the mycelia were exposed to high concentrations of H_2_O_2_ (8 and 12 mM); this may be caused by a species difference or by the different treatment approaches. For example, to make *sop1* expression correspond to the growth of the wild-type strain and the *sop1* silenced transformants of *S. sclerotiorum* on CM medium supplemented with different concentrations of H_2_O_2_, the mycelia were cultured for 24 h for qRT-PCR analysis. The *sop1* silenced transformants had a pleiotropic phenotype, indicating that *sop1* has diverse biological functions, which is obviously different from its homologs in many filamentous fungi. Our results indicated that biological functions of microbial opsins may be not conserved in different organisms.

Previous research indicated obvious cross-talk between the osmotic stress signaling pathway and the oxidative stress pathway. For examples, the stress-activated MAP kinase BcSak1 could be activated by both oxidative and osmotic stress. Additionally, several genes were induced by oxidative stress treatment and most of them were also induced by osmotic stress (Heller et al., 2012). The Hog1 homologs are responsible not only for osmoregulation but also for oxidative stress response in many filamentous fungi (Dixon et al., 1999; Zhang et al., 2002; Du et al., 2006; Moriwaki et al., 2006). In this study, we noted the novel example that sop1 also responded to both oxidative and osmotic stress, although in a contrary manner: the presence of sop1 makes *S. sclerotiorum* more sensitive to oxidative stress, but more tolerant to osmotic stress. Large quantities of ROS are harmful for both pathogens and plant cells. In plants, the rapid accumulation of ROS can directly kill the cells surrounding the infection site. Local plant cell death caused by the hypersensitive response (HR) restricts biotrophic pathogens and inhibits their further expansion into neighbor cells and consequently protects the other parts of the plant (Talarczyk and Hennig, 2001). The oxidative burst is an effective process that protects the host against biotrophic pathogens that depend on living host cells but not necrotrophs that live on dead plant tissues (Rivas and Thomas, 2005). On the contrary, the HR reaction facilitates plant infection by necrotrophic pathogens (Govrin and Levine, 2000). For pathogens, higher levels of ROS evoke oxidative stress to harm the pathogen directly, so ROS tolerance is crucial for pathogens to eliminate ROS in their own cells with ROS-detoxification systems such as catalases, peroxidases or superoxide dismutase (SOD) (Temme and Tudzynski, 2009). A previous research showed that *B. cinerea* induces a significant oxidative burst in all host tissues analyzed (Lyon et al., 2007) but does not suffer H_2_O_2_-induced oxidative stress in plants (Temme and Tudzynski, 2009). In this study, our results showed that high concentration of H_2_O_2_ down-regulates *sop1* expression in *S. sclerotiorum* and low *sop1* expression level in turn increases oxidative stress tolerance. This is the first report of a novel positive feedback mechanism that allows *S. sclerotiorum* to strengthen its tolerance to oxidative stress through the regulation of *sop1* expression.

Additionally, as a predicted conserved cytomembrane protein necessary for full virulence, normal growth and sclerotial development of *S. sclerotiorum*, sop1 homologs are only present in the microbes and lower plants, but not in higher plants and animals (with a single exception as previously noted). These properties make sop1 a potential drug target for *S. sclerotiorum*. Furthermore, our results indicated that the possibility of generation of fungicide-resistance or tolerance existed in the wild-type strain of *S. sclerotiorum* (Figures 4H–J), however disabling sop1 makes *S. sclerotiorum* more sensitive to fungicides and to various extreme environments, indicating that sop1 is a prospective, attractive drug target because it not necessary to consider fungicide-resistance or tolerance in the control of this pathogen.

## Conclusion

The microbial opsin homolog sop1 was experimentally confirmed to be essential for growth, sclerotial development and full virulence of *S. sclerotiorum*. *Sop1* plays many roles in responding to multiple environmental signals such as osmotic and oxidative stresses and is involved in fungicide resistance, which may be responsible for the environmental adaptability of *S. sclerotiorum*. Furthermore, our results showed that microbial opsins do not exist in animals and almost all higher plants indicating that sop1 is a potential novel drug target for disease control of *S. sclerotiorum*.

## Author contributions

XL and JC designed the research and wrote the paper; XL and CS executed the experiments. XL, JX, YF, DJ, GL, and JC performed the data and bioinformatics analyses. All authors read and approved the final manuscript.

### Conflict of interest statement

The authors declare that the research was conducted in the absence of any commercial or financial relationships that could be construed as a potential conflict of interest.

## Footnotes

^1^http://blast.ncbi.nlm.nih.gov/Blast.cgi?PROGRAM=blastp&PAGE_TYPE=BlastSearch&LINK_LOC=blasthome.

## Supplementary material

Figure S1 *****Sop1*** silenced transformants showing significantly reduced virulence on detached tomato leaves**. Virulence of the *sop1* silenced transformants and the wild-type strain was evaluated according to the lesion diameter at 20°C for 48 h. Three independent replications were performed. The values are presented as the mean ± s.d. Differentiation was evaluated by a *t*-test. Different letters on the graph indicate statistical significance, *P* = 0.05.

Figure S2 **Biological characterization of the ***sop1*** silenced transformants obtained by the other gene silencing strategy. (A)** The construction of *sop1* silencing vector pRNAi-2. **(B)** Phenotypes of the *sop1* silenced transformants and the wild-type strain grown on PDA at 20°C. Photos were taken at 8 dpi. **(C)** Comparison of the virulence of the silenced transformants and the wild-type strain on detached oilseed rape leaves at 20°C for 48 h. **(D)** Relative *sop1* expression level of the *sop1* silenced transformants and the wild-type strain as determined by qRT-PCR analysis. A value of 1 was assigned to the abundance of cDNA from the wild-type strain. The *sop1* expression levels in the silenced transformants and the wild-type strain were normalized to that of the β-tubulin transcripts from each strain. **(E)** Comparison of lesion diameter of the silenced transformants and the wild-type strain. **(F)** Comparison of the hyphal growth rate of the silenced transformants and the wild-type strain. Three independent replications were performed. Bars indicate the standard error. The values are presented as the mean ± s.d. Differentiation was evaluated by a *t*-test. Different letters on a graph indicate statistical significance, *P* = 0.05.

Table S1 **Differentially expressed signaling genes identified by the DGE during different developmental stages of ***S. sclerotiorum*** compared with the vegetative growth stage**. Red and green indicate significantly up-regulated and down-regulated gene expression, respectively. Blank cells without numbers indicate genes that were not detected in the DGE data or not differentially expressed during corresponding developmental stages.
