# Supplementary figures and images for: The Microbial Opsin Homolog Sop1 is involved in Sclerotinia sclerotiorum Development and Environmental Stress Response
Source: Front Microbiol. 2016 Jan 7;6:1504. doi: 10.3389/fmicb.2015.01504 (PMC4703900; doi:10.3389/fmicb.2015.01504)

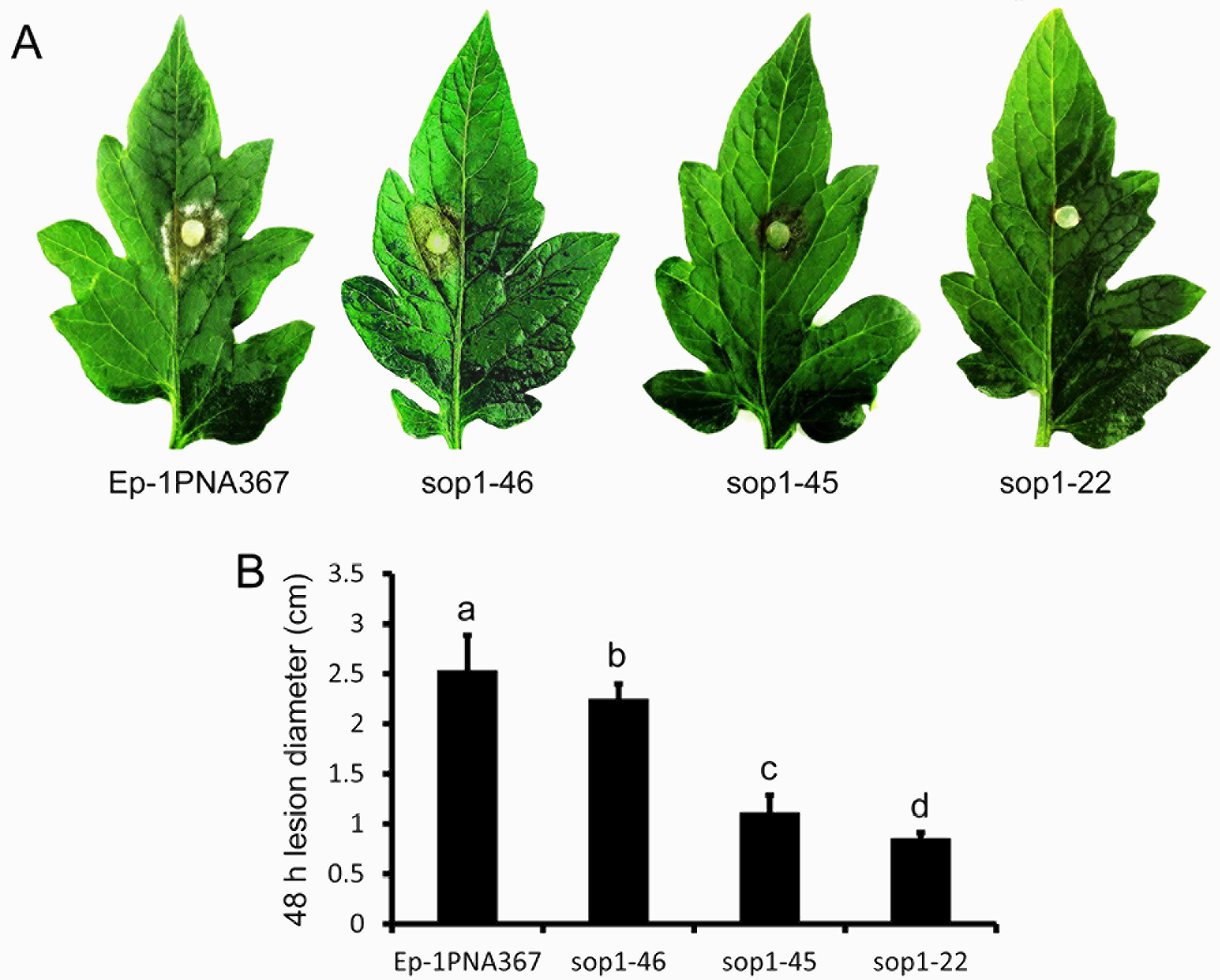

Supplement: Figure S1 — Sop1 silenced transformants showing significantly reduced virulence on detached tomato leaves. Virulence of the sop1 silenced transformants and the wild-type strain was evaluated according to the lesion diameter at 20°C for 48 h. Three independent replications were performed. The values are presented as the mean ± s.d. Differentiation was evaluated by a t-test. Different letters on the graph indicate statistical significance, P = 0.05. [file Image1.TIFF]

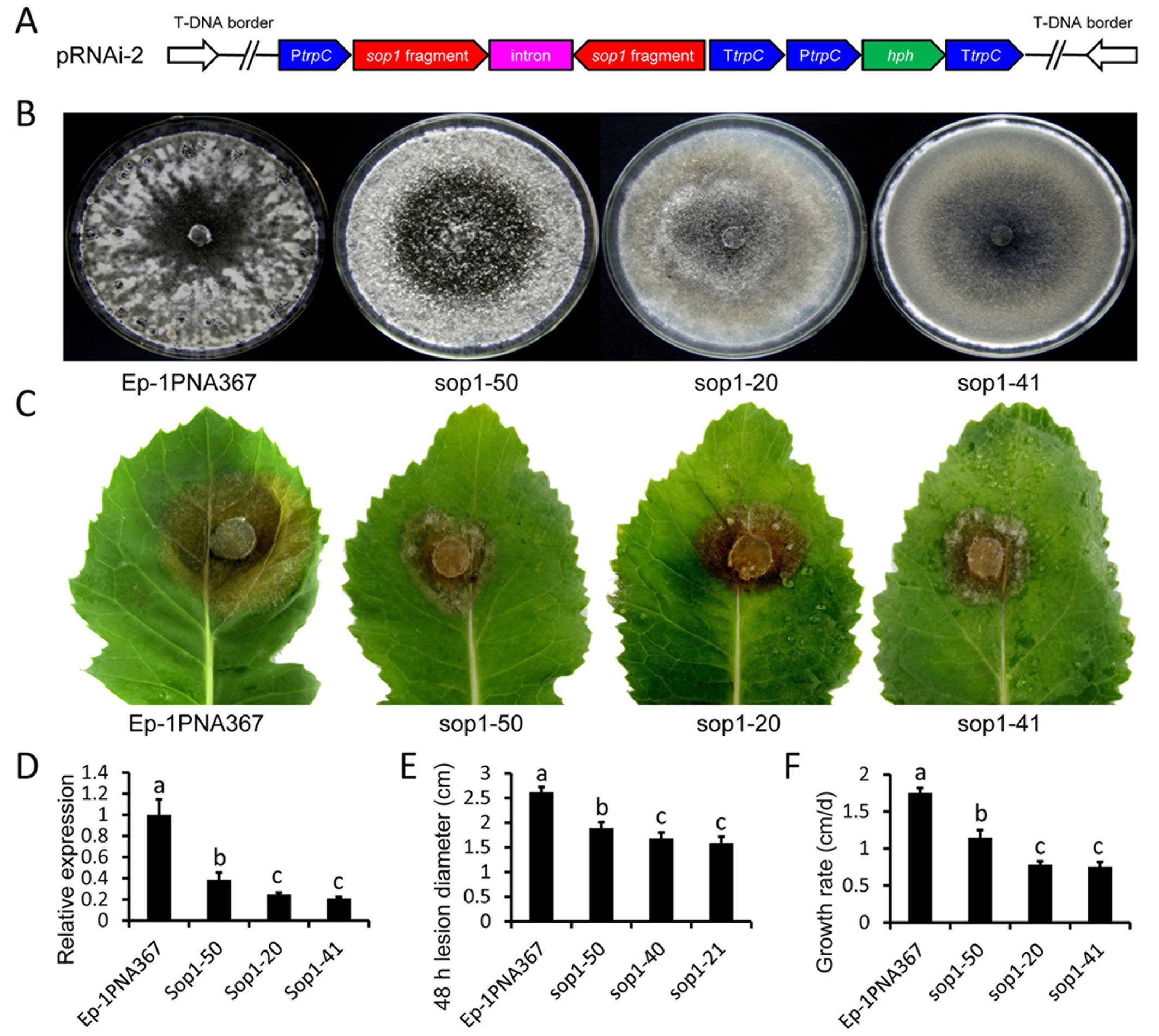

Supplement: Figure S2 — Biological characterization of the sop1 silenced transformants obtained by the other gene silencing strategy. (A) The construction of sop1 silencing vector pRNAi-2. (B) Phenotypes of the sop1 silenced transformants and the wild-type strain grown on PDA at 20°C. Photos were taken at 8 dpi. (C) Comparison of the virulence of the silenced transformants and the wild-type strain on detached oilseed rape leaves at 20°C for 48 h. (D) Relative sop1 expression level of the sop1 silenced transformants and the wild-type strain as determined by qRT-PCR analysis. A value of 1 was assigned to the abundance of cDNA from the wild-type strain. The sop1 expression levels in the silenced transformants and the wild-type strain were normalized to that of the β-tubulin transcripts from each strain. (E) Comparison of lesion diameter of the silenced transformants and the wild-type strain. (F) Comparison of the hyphal growth rate of the silenced transformants and the wild-type strain. Three independent replications were performed. Bars indicate the standard error. The values are presented as the mean ± s.d. Differentiation was evaluated by a t-test. Different letters on a graph indicate statistical significance, P = 0.05. [file Image2.TIFF]
